# BRD4 Inhibition Suppresses Senescence and Apoptosis of Nucleus Pulposus Cells by Inducing Autophagy during Intervertebral Disc Degeneration: An In Vitro and In Vivo Study

**DOI:** 10.1155/2022/9181412

**Published:** 2022-03-11

**Authors:** Guang-Zhi Zhang, Hai-Wei Chen, Ya-jun Deng, Ming-Qiang Liu, Zuo-Long Wu, Zhan-Jun Ma, Xue-Gang He, Yi-Cheng Gao, Xue-Wen Kang

**Affiliations:** ^1^Department of Orthopedics, Lanzhou University Second Hospital, Lanzhou, Gansu 730000, China; ^2^The Second Clinical Medical College, Lanzhou University, Lanzhou, Gansu 730000, China; ^3^Key Laboratory of Orthopedics Disease of Gansu Province, Lanzhou University Second Hospital, Lanzhou, Gansu Province 730030, China; ^4^The International Cooperation Base of Gansu Province for the Pain Research in Spinal Disorders, Lanzhou, Gansu Province 730030, China

## Abstract

Intervertebral disc degeneration (IDD) is the most common chronic skeletal muscle degeneration disease. Although the underlying mechanisms remain unclear, nucleus pulposus (NP) autophagy, senescence, and apoptosis are known to play a critical role in this process. Previous studies suggest that bromodomain-containing protein 4 (BRD4) promotes senescent and apoptotic effects in several age-related degenerative diseases. It is not known, however, if BRD4 inhibition is protective in IDD. In this study, we explored whether BRD4 influenced IDD. In human clinical specimens, the BRD4 level was markedly increased with the increasing Pfirrmann grade. At the cellular level, BRD4 inhibition prevented IL-1*β*-induced senescence and apoptosis of NP cells and activated autophagy via the AMPK/mTOR/ULK1 signaling pathway. Inhibition of autophagy by 3-methyladenine (3-MA) partially reversed the antisenescence and antiapoptotic effects of BRD4. In vivo, BRD4 inhibition attenuated IDD. Taken together, the results of this study showed that BRD4 inhibition reduced NP cell senescence and apoptosis by induced autophagy, which ultimately alleviated IDD. Therefore, BRD4 may serve as a novel potential therapeutic target for the treatment of IDD.

## 1. Introduction

Low back pain is a common symptom of intervertebral disc (IVD) degeneration (IDD) and the most common cause of loss of work ability and reduced quality of life, imposing a considerable economic burden on the family and society [1–3]. Epidemiological studies have shown that about 40% of individuals <30 years of age suffer from IDD, and this increases to more than 90% in those >50 years of age [4]. IVD degeneration, if allowed to progress unchecked, leads to spinal diseases such as herniated disc, spondylolisthesis, and spinal stenosis, resulting in acute or chronic low back pain [1]. A variety of internal and external factors—e.g., genetic susceptibility, mechanical pressure, lifting heavy loads, smoking, obesity, and aging—are linked to the development of IDD [5–7]. In the pathogenesis of IDD, the water content of nucleus pulposus (NP) tissue gradually decreases, large amounts of proteoglycan and collagen II are lost, and disrupted extracellular matrix (ECM) metabolism leads to loss of the function and structure of the IVD [8]. Although the physiology and pathology of IDD are established, the underlying molecular mechanism is unclear.

The IVD has three parts: the central NP, the peripheral fibrous annulus (AF), and the cartilage endplates (CEP) on both sides. The NP is a translucent jelly-like tissue and is the central component of the IVD. It is rich in ECM components such as collagen II and aggrecan; due to its higher water content, it has marked elasticity and antitension properties, maintaining the stability and physiological curvature of the spine [9]. IDD is a complex cell-mediated process involving senescence and apoptosis of NP cells, leading to changes in NP function and structure [3]. Senescence reduces the number of functional cells, accelerates the senescence of neighbouring cells, and enhances inflammation and ECM catabolism [10, 11]. Apoptosis accelerates IDD by reducing the number of living cells [12]. Inhibiting the senescence and apoptosis of NP cells has a protective effect on IDD [10, 13]. Autophagy is related to degenerative diseases (e.g., osteoarthritis, neurodegenerative diseases, and cancer) [14, 15], and is a protective mechanism activated by NP cells to suppress senescence and apoptosis. Activation of autophagy also alleviates IDD [16]. Therefore, studying the crosstalk of autophagy, senescence, and apoptosis in NP cells will provide insight into the pathogenesis of, and facilitate identification of potential therapeutic targets for, IDD.

Bromodomain-containing protein 4 (BRD4), a member of the bromodomain and ultra-terminal structure family, regulates gene transcription and the cell cycle and inflammatory response [17]. BRD4 is linked to a variety of aging-related diseases, such as ischemic brain injury [17], cardiovascular disease [18], pulmonary fibrosis [19], diabetes [20], and cancer [21]. Jiang et al. [22] reported that BRD4 is upregulated in the articular cartilage of patients with osteoarthritis and that it has therapeutic potential. An et al. [23] showed that BRD4 expression was significantly increased by H_2_O_2_ in rat chondrocytes, and BRD4 inhibition prevented oxidative stress-mediated apoptosis and cartilage matrix degeneration. However, to our knowledge, no *in vitro* or *in vivo* study has yet evaluated the relationships between BRD4 status, senescence and apoptosis during IDD.

We report herein that BRD4 expression in human NP tissues increases with IDD severity. BRD4 inhibition delays IDD by regulating the AMP-activated protein kinase (AMPK)/mammalian target of rapamycin (mTOR)/Unc-51-like autophagy-activating kinase (ULK1) signaling pathway to activate autophagy and inhibit the senescence and apoptosis of NP cells. The rat annulus needle-puncture model confirmed the protective effect of BRD4 inhibition on IDD *in vivo*. Therefore, BRD4 has therapeutic potential for preventing the progression of IDD.

## 2. Results

### 2.1. BRD4 Expression Is Increased in Degenerated Human IVD NP Tissue

**Figure 1:**
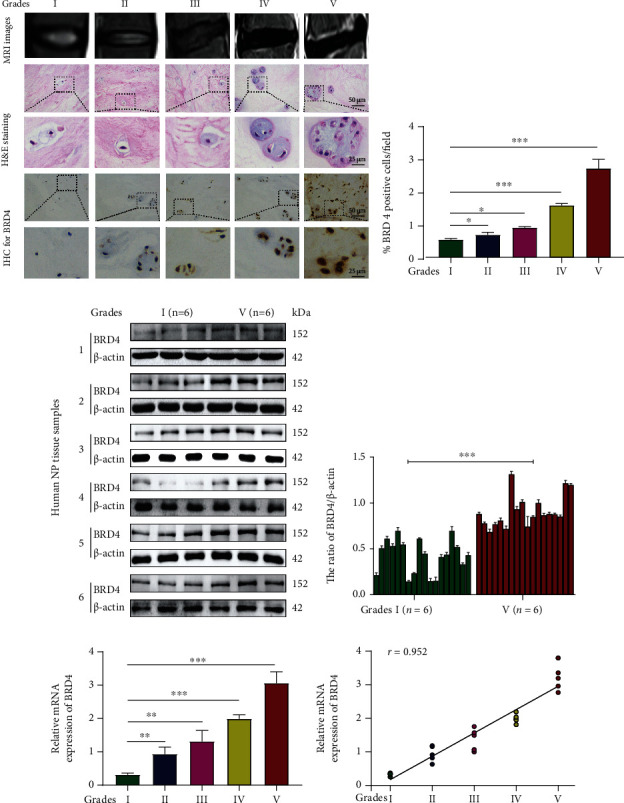
BRD4 expression is increased in degenerated human IVD NP tissue. (a) Representative magnetic resonance imaging (MRI), haematoxylin-eosin- (HE-) stained, and immunohistochemical (IHC) images of degenerated human IVDs (grades I to V). (b) Quantification of BRD4 protein level in NP tissues according to the Pfirrmann grade by IHC. (c, d) Representative western blot and quantification data of BRD4 in NP tissues by the Pfirrmann grade. (e) BRD4 mRNA expression in different groups as shown by real-time RT-PCR. (f) Correlation of the BRD4 mRNA level with the Pfirrmann grade of NP tissues by nonparametric linear regression. Columns are means ± SD. Significant differences between the treatment and control groups: ^∗^*P* < 0.05, ^∗∗^*P* < 0.01, and ^∗∗∗^*P* < 0.001.

To evaluate its relationship with IDD progression, we evaluated BRD4 expression in NP tissues of different degrees of degeneration. The Pfirrmann grade I to V groups of NP tissues each comprised six samples. Representative magnetic resonance images (MRI) of the degenerated IVDs are shown in Figure 1(a). Haematoxylin-eosin (HE) staining showed that the characteristics of NP tissue changed with the severity of degeneration. In the grade I and II groups, the collagen fibres in NP tissue were arranged neatly, the ECM of NP cells was homogeneously and deeply stained and a few small vacuolar cells were uniformly distributed. In the grade III group, the collagen fibres were thickened and irregular and there were more aggregations in the NP area. In the grade IV and V groups, the collagen fibres in NP tissue were highly disordered and NP cells aggregated in large numbers, appearing as large vacuolar cells (Figure 1(a)). Immunohistochemistry (IHC) demonstrated that the BRD4 protein level in NP tissue increased with increasing Pfirrmann grade (Figures 1(a) and 1(b)). The levels of BRD4 protein in six grade I and six grade V NP tissue samples were evaluated by western blotting. Compared with that in grade I NP tissues, BRD4 expression was significantly increased in grade V NP tissues (Figures 1(c) and 1(d)). Finally, the levels of BRD4 mRNA were evaluated by RT-PCR, the result also show that BRD4 mRNA level in NP was positively correlated with the Pfirrmann grade (*n* = 36; *r* = 0.952, *P* < 0.001) (Figures 1(e) and 1(f)).

### 2.2. Identification of Human Primary NP Cells in Culture

Specific markers of NP cells are lacking, and these cells are instead identified based on their chondrocyte-like characteristics [24, 25]. By optical microscopy, human primary NP cells were long-fusiform, polygonal, irregularly, or star shaped (Supplementary Figure 1A, 1B, C). HE staining showed that human NP cells were of short-spindle, polygonal or other irregular shapes (Supplementary Figure 1D). Safranin-O stained the nuclei of NP cells dark red, with light staining of the patina (Supplementary Figure 1E). Toluidine blue stained the proteoglycans in NP cells indigo, with the nucleus located in the centre of the cell or off to one side (Supplementary Figure 1F). Immunofluorescence staining showed extensive collagen II, Sox9, and aggrecan fluorescence in the cytoplasm, which increased with increasing proximity to the blue-stained nucleus (Supplementary Figures 1G, 1H, and 1I).

### 2.3. BRD4 Expression Increased in Senescent NP Cells

**Figure 2:**
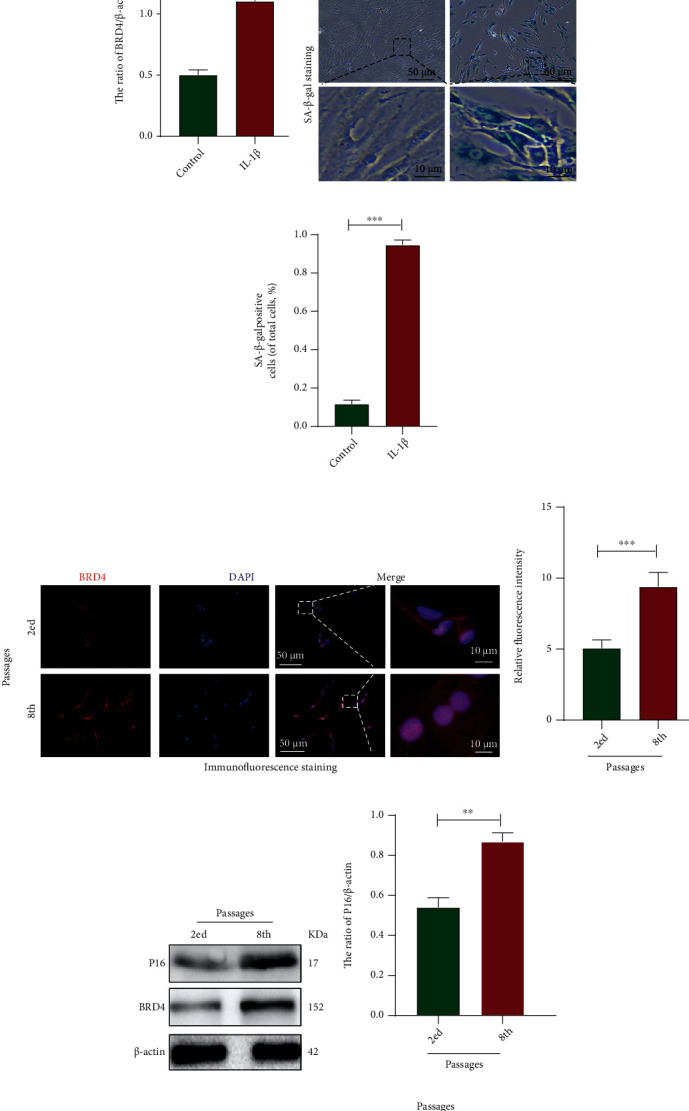
BRD4 expression increased in senescent NP cells. (a, b) Representative immunofluorescence and quantification of BRD4 in the control and IL-1*β* groups. (c–e) Representative western blots and quantification of BRD4 in the control and IL-1*β* groups. (f, g) Representative SA-*β*-gal staining and quantification of SA-*β*-gal activity in the control and IL-1*β* groups. (h, i) Representative immunofluorescence and quantification of BRD4 in the NP cells at passages 2 and 8. (j–l) Representative western blots and quantification of BRD4 at passages 2 and 8. (m, n) Representative SA-*β*-gal staining and quantification of SA-*β*-gal activity in the NP cells at passages 2 and 8. Columns are means ± SD. Significant differences between the treatment and control groups: ^∗^*P* < 0.05, ^∗∗^*P* < 0.01, and ^∗∗∗^*P* < 0.001.

As IDD progresses, the levels of inflammatory factors in IVDs increase significantly, including interleukin- (IL-) 1*α*, IL-1*β*, IL-6, IL-17, and tumour necrosis factor- (TNF-) *α*. These induce local autoimmune inflammation and cause metabolic disorders of ECM. Increased catabolism leads to dysfunction and structural changes of the IVD, leading to IDD [3]. A model of IL-1*β*-induced NP cells senescence has been used in studies of IDD [13]. Immunofluorescence, western blotting, and SA-*β*-gal staining showed that IL-1*β* significantly increased the levels of P16 (indicator of senescence) and BRD4 in NP cells (Figures 2(a)–2(e)), as well as the SA-*β*-gal activity (Figures 2(f) and 2(g)). Therefore, the BRD4 level is elevated in senescent NP cells.

IDD is linked to senescence, an important type of which is replicative senescence [13]. Immunofluorescence and western blotting showed that the level of BRD4 in passage-8 NP cells was significantly increased compared to that in passage-2 NP cells (Figures 2(h) and 2(i)). Also, P16 and SA-*β*-gal activity were assayed to assess NP cells senescence. The P16 level and SA-*β*-gal activity increased as the passage number increased (replication senescence) (Figures 2(j)–2(n)). Therefore, the BRD4 level increases as cells enter senescence.

Therefore, the expression of BRD4 in NP cells was increased by senescence, suggesting that BRD4 plays a key role in the senescence of NP cells.

### 2.4. BRD4 Inhibition Reduces IL-1*β*-Induced Senescence and Apoptosis of Human NP Cells

**Figure 3:**
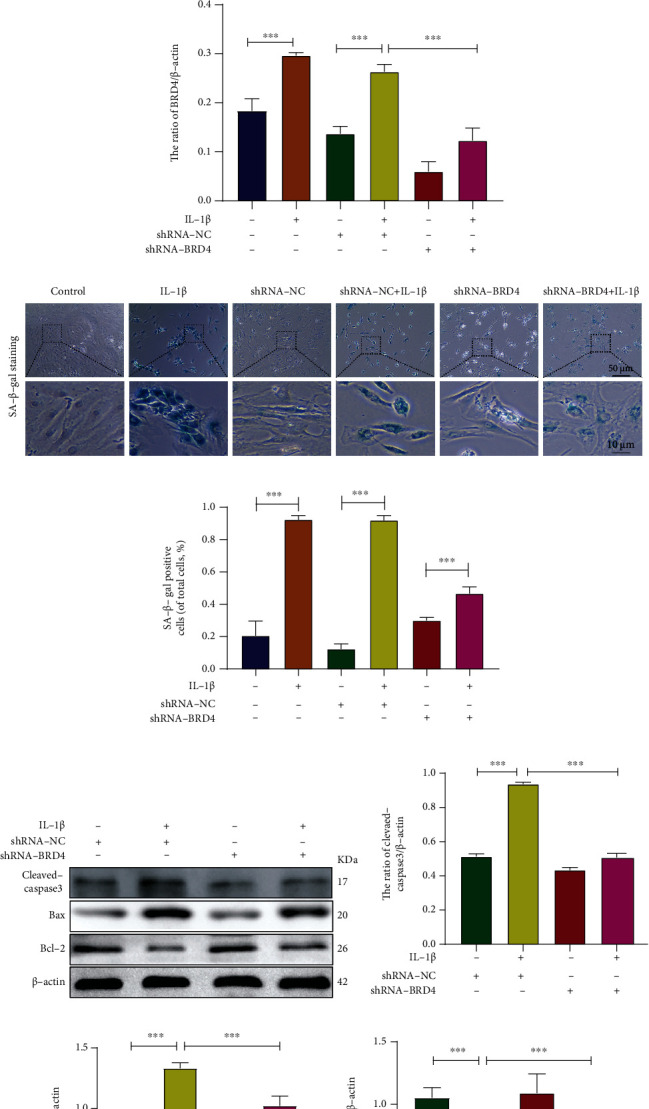
BRD4 inhibition reduces IL-1*β*-induced senescence of human NP cells. (a, b) Representative western blots and quantification of BRD4 in the shRNA-NC and shRNA-BRD4 groups. (c) RT-PCR showed that the BRD4 mRNA level in the shRNA-NC and shRNA-BRD4 groups. (d–g) Representative western blots and quantification of P16, P21, and BRD4 in human NP cells. (h, i) Representative SA-*β*-gal staining and quantification of SA-*β*-gal activity in the NP cells; blue, senescent NP cells. (j–m) Representative western blots and quantification of cleaved caspase-3, Bcl-2, and Bax in human NP cells. (n–o) Representative Hoechst 33258-stained fluorescence images and quantification of the proportion of apoptotic cells (white arrows, apoptotic cells).. Columns are means ± SD. Significant differences between the treatment and control groups: ^∗^*P* < 0.05, ^∗∗^*P* < 0.01, and ^∗∗∗^*P* < 0.001.

BRD4 expression was increased in senescent NP cells. NP cells were infected with lentiviruses expressing scrambled shRNA (shRNA-NC) and BRD4 shRNA (shRNA-BRD4), and evaluated silencing efficiency at the mRNA and protein levels (Figures 3(a)–3(c)). Western blotting showed that, compared to the control and shRNA groups, the P16, P21, and BRD4 levels were significantly decreased in the shRNA-BRD4 group irrespective of IL-1*β* (Figures 3(d)–3(g)). SA-*β*-gal activity was significantly increased by IL-1*β* and was significantly reduced by BRD4 inhibition (Figures 3(h) and 3(i)). In summary, in the presence of IL-1*β*, inhibition of BRD4 significantly reduced NP cells senescence.

We next evaluated whether inhibition of BRD4 suppresses IL-1*β*-induced apoptosis of human NP cells. The levels of cleaved caspase-3 and Bax increased during apoptosis. Bcl-2 inhibits apoptosis, and its level decreases as apoptosis increases [26]. Compared with the shRNA-NC group, IL-1*β* increased the levels of Bax and cleaved caspase-3, but their levels were significantly reduced in the shRNA-BRD4 group. Inhibition of BRD4 significantly increased the Bcl-2 level (Figures 3(j)–3(m)). Hoechst 33258 staining showed that compared with the shRNA-NC group, the shRNA-BRD4 group showed fewer apoptotic cells irrespective of the presence of IL-1*β* (Figures 3(n) and 3(o)). Therefore, BRD4 inhibition suppresses IL-1*β*-induced apoptosis of human NP cells.

### 2.5. BRD4 Inhibition Enhances Autophagy via the AMPK/mTOR/ULK1 Signaling Pathway in Human NP Cells

**Figure 4:**
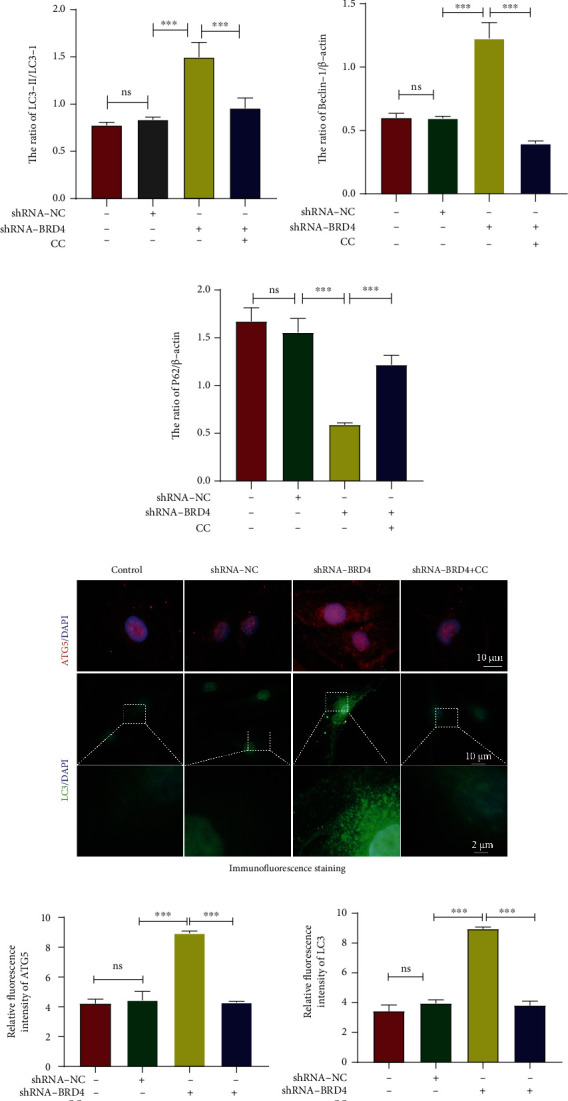
BRD4 inhibition enhances autophagy via the AMPK/mTOR/ULK1 signaling pathway in human NP cells. (a–g) Representative western blots and quantification of AMPK, p-AMPK, mTOR, p-mTOR, ULK1, p-ULK1, LC3, Beclin-1, and P62 protein levels in human NP cells. (h–j) Representative immunofluorescence and quantification of ATG5 (red) and LC3 (green) in NP cells treated as above. (k, l) Representative transmission electron microscopy and quantification of double-membrane autophagosomes in NP cells after shRNA-BRD4 treatment. Columns are means ± SD. Significant differences between the treatment and control groups: ^∗^*P* < 0.05, ^∗∗^*P* < 0.01, and ^∗∗∗^*P* < 0.001.

To explore the effect of BRD4 inhibition on autophagy of human NP cells, we evaluated the LC3-II/LC3-I ratio and the Beclin-1 and P62 levels (autophagy markers) by western blotting. The LC3-II/LC3-I ratio and Beclin-1 level in the shRNA-BRD4 group were higher than those in the shRNA-NC group, whereas the P62 level was lower (Figures 4(a) and 4(e)–4(g)). Immunofluorescence showed that the expression of ATG5 in NP cells increased significantly after BRD4 inhibition, as did the number of LC3-positive spots (autophagic vesicles) (Figures 4(h)–4(j)). TEM showed that the number of autophagosomes increased in the shRNA-BRD4 group, indicating a key role for autophagy (Figures 4(k) and 4(l)). In conclusion, BRD4 inhibition activated autophagy in human NP cells.

To identify the signaling pathway(s) responsible for autophagy activation after BRD4 inhibition, we assayed the levels of AMPK, mTOR (downstream target of AMPK), and ULK1 (downstream target of mTOR complex 1), as well as the changes therein. Western blotting showed that the levels of p-AMPK and p-ULK1 were increased by BRD4 inhibition, whereas that of p-mTOR decreased. To investigate the role of the AMPK/mTOR/ULK1 signaling pathway in the autophagy activation induced by BRD4 inhibition, NP cells were preincubated with the AMPK inhibitor compound C (CC, 10 *μ*M) 24 h before shRNA-BRD4 treatment. Compared with NP cells treated with shRNA-BRD4 alone, application of CC significantly reversed the increase in the p-AMPK and p-ULK1 levels and the decrease in the p-mTOR level (Figures 4(a)–4(d)). Also, CC, by inhibiting the AMPK/mTOR/ULK1 signaling pathway, significantly reduced the level of shRNA-BRD4-mediated activation of autophagy (Figures 4(a) and 4(e)–4(g)). Immunofluorescence and TEM confirmed this effect (Figures 4(h) and 4(l)). Therefore, the AMPK/mTOR/ULK signaling pathway is involved in autophagy activation triggered by inhibition of BRD4.

### 2.6. Inhibition of Autophagy Reduces the Antisenescence and Antiapoptotic Effect of BRD4

**Figure 5:**
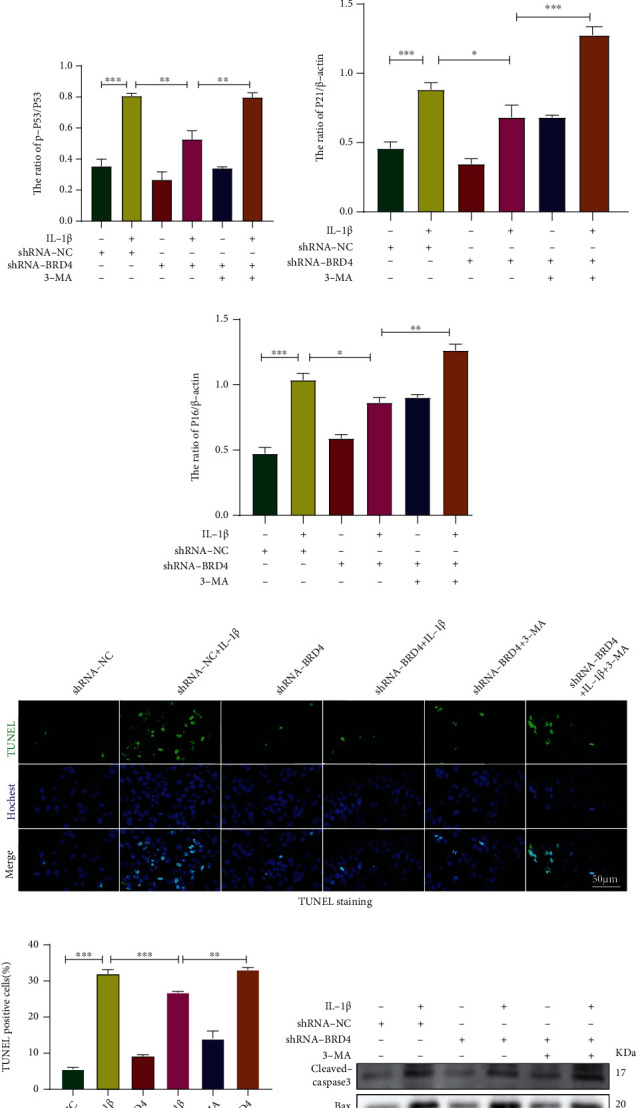
Inhibition of autophagy reduces the antisenescence and antiapoptotic effect of BRD4. (a, b) Representative SA-*β*-gal staining and quantification of SA-*β*-gal activity in the NP cells; blue, senescent NP cells. (c–f) Representative western blots and quantification of P16, P21, P53, and p-P53 in human NP cells. (g) Representative TUNEL images. Nuclei were stained with Hoechst (blue), and apoptotic cells were visualised by fluorescence microscopy. (h) Proportion of apoptotic cells by TUNEL staining. (i–l) Representative western blots and quantification of cleaved caspase-3, Bcl-2, and Bax levels in human NP cells. Columns are means ± SD. Significant differences between the treatment and control groups: ^∗^*P* < 0.05, ^∗∗^*P* < 0.01, and ^∗∗∗^*P* < 0.001.

To determine whether autophagy is involved in the antisenescence effect of BRD4, we pretreated NP cells with the classic autophagy inhibitor 3-methyladenine (3-MA). Compared to the shRNA-NC group, the SA-*β*-gal activity in the shRNA-NC+IL-1*β* group was significantly increased; shRNA-BRD4 reversed this effect. However, 3-MA eliminated the antisenescence effect of BRD4 by inhibiting autophagy (Figures 5(a) and 5(b)). Similarly, western blotting showed that BRD4 inhibition significantly reduced the IL-1*β*-induced P16, P21 and p-P53 (cyclin-dependent kinase inhibitors, a marker of cell senescence) expression levels. Also, 3-MA significantly reduced the antisenescence effect of BRD4 (Figures 5(c)–5(f)). Therefore, BRD4 inhibition regulates NP cells senescence by mediating autophagy.

To determine whether autophagy is involved in the antiapoptotic effect of BRD4, we pretreated NP cells with 3-MA. TUNEL staining results showed that compared with the shRNA-NC group, the shRNA-NC+IL-1*β* group showed a larger number of apoptotic cells. However, BRD4 inhibition significantly reduced NP cells apoptosis, an effect reversed by 3-MA (Figures 5(g) and 5(h)). BRD4 inhibition significantly reduced the IL-1*β*-induced upregulation of cleaved caspase-3 and Bax and increased that of the antiapoptotic factor Bcl-2. Compared with the shRNA-BRD4+IL-1*β* group, the shRNA-BRD4+IL-1*β*+3-MA group showed increased levels of cleaved caspase-3 and Bax, whereas 3-MA significantly reduced the level of Bcl-2 (Figures 5(i)–5(l)). Therefore, the antiapoptotic effect of BRD4 is mediated by enhancement of autophagy.

### 2.7. BRD4 Inhibition Modulates the ECM Protein Level by Mediating Autophagy in NP Cells

**Figure 6:**
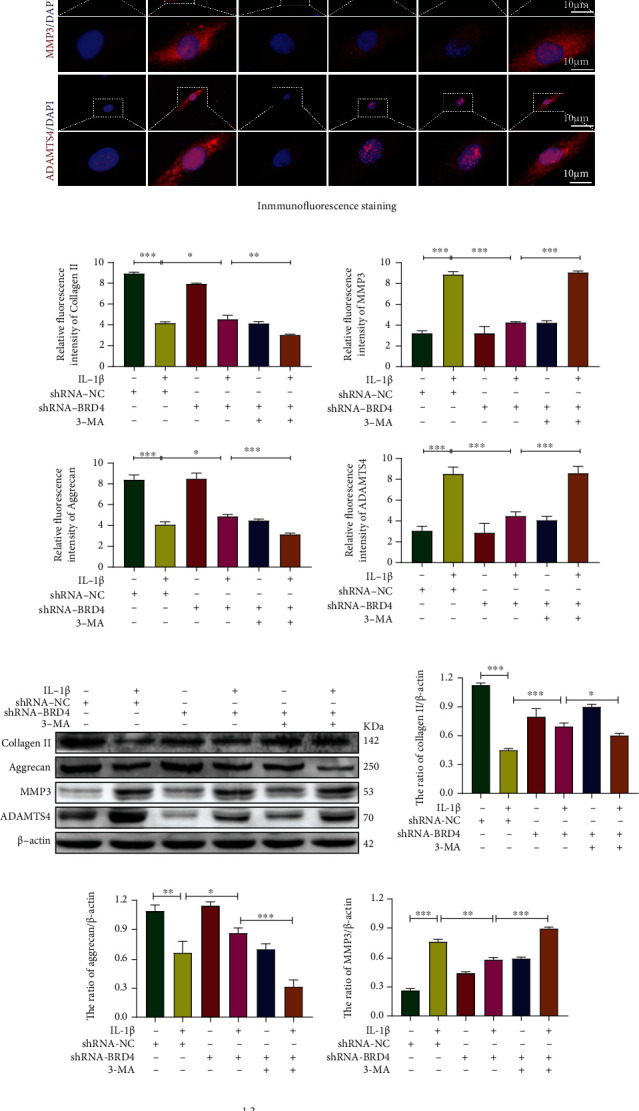
BRD4 inhibition modulates ECM protein levels by mediating autophagy in NP cells. (a, b) Representative immunofluorescence and quantification of collagen II (green), aggrecan (green), MMP3 (red), and ADAMTS4 (red) in NP cells. (c–g) Representative western blots and quantification of collagen II, aggrecan, MMP3, and ADAMTS4 in human NP cells. Columns are means ± SD. Significant differences between the treatment and control groups: ^∗^*P* < 0.05, ^∗∗^*P* < 0.01, and ^∗∗∗^*P* < 0.001.

The ECM protein composition reflects the functional status of NP cells. NP tissue is rich in ECM components—such as collagen II, elastin, and aggrecan—which are essential for maintaining the gel properties of NP tissue and to offset and transmit axial pressure loads during spine stress [1]. To investigate whether autophagy is involved in BRD4-mediated ECM catabolism, we assessed the levels of major ECM proteins (collagen II and aggrecan) and matrix-degrading enzymes (MMP3 and ADAMTS4) in human NP cells. Immunofluorescence showed that, in the presence of IL-1*β*, the levels of collagen II and aggrecan decreased significantly and those of MMP3 and ADAMTS4 increased significantly. BRD4 inhibition significantly attenuated these effects of IL-1*β*. Importantly, compared with the shRNA-BRD4+IL-1*β* group, the levels of MMP3 and ADAMTS4 in the shRNA-BRD4+IL-1*β*+3-MA group increased significantly, and those of collagen II and aggrecan decreased significantly (Figures 6(a) and 6(b)). The western blot results (Figures 6(c)–6(g)) were consistent with the immunofluorescence results. Thus viewed, the ECM is weakened by anabolism and strengthened by catabolism after induction by IL-1*β*. Inhibition of BRD4 decreases ECM catabolism and increases its metabolism; however, 3-MA inhibited this effect. Therefore, by regulating autophagy BRD4 ameliorates the effects of metabolic disorders on the ECM.

### 2.8. BRD4 Inhibition Ameliorates IDD Induced by Annulus Needle Puncture *In Vivo*

**Figure 7:**
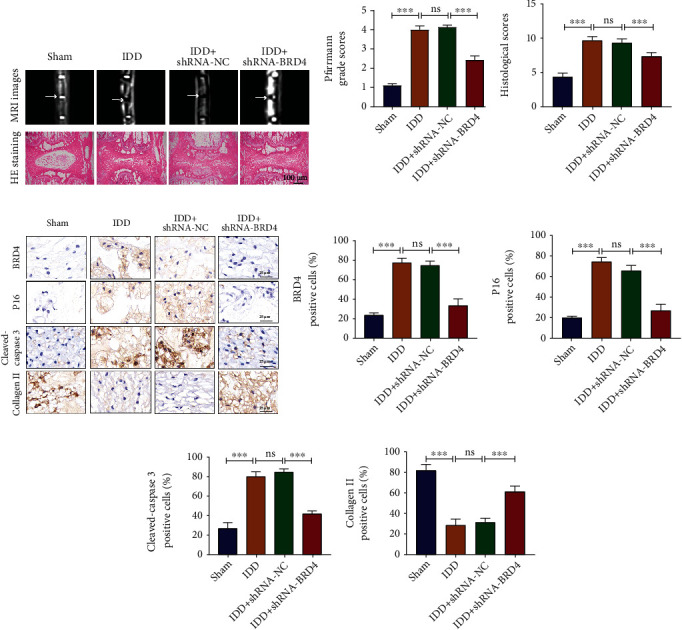
BRD4 inhibition reduces IDD induced by annulus-needle puncture *in vivo*. (a, b) Representative T2-weighted images and HE staining of a needle-punctured disc at 8 weeks (white arrows). (b, c) Pfirrmann grades and histological scores of a needle-punctured disc at 8 weeks. (d–h) IHC staining and quantitative analysis of BRD4, P16, cleaved caspase-3, and collagen II in disc samples 8 weeks after puncture. Columns are means ± SD. Significant differences between the treatment and control groups: ^∗^*P* < 0.05, ^∗∗^*P* < 0.01, and ^∗∗∗^*P* < 0.001.

To assess the therapeutic effect of shRNA-BRD4 on fine-needle aspiration-induced IDD *in vivo*, we used the rat annulus-needle puncture model of IDD. SD rats were randomly assigned into the sham, IDD, IDD+shRNA-NC, and IDD+shRNA-BRD4 groups (*n* = 24). Except for those in the sham group, rats were injected with phosphate-buffered saline (PBS), shRNA-NC, or shRNA-BRD4. At 8 weeks after puncture, the IVD T2-weighted signal intensity of the IDD+shRNA-BRD4 group was higher than that in the IDD and IDD+shRNA-NC groups. Compared with the IDD and IDD+shRNA-NC groups, the IDD+shRNA-BRD4 group had a lower Pfirrmann score (Figures 7(a) and 7(b)). With shRNA-BRD4 treatment, these degenerative changes of the disc structure were alleviated. Histological scores also indicated that shRNA-BRD4 protected against IDD development (Figures 7(a)–7(c)). IHC analysis showed that the level of BRD4 in the IDD+shRNA-BRD4 group was significantly decreased compared with that in the sham, IDD, and IDD+shRNA-NC groups. This verified successful transfection by IVD injection of shRNA-BRD4. Importantly, IHC staining of P16, cleaved caspase-3, and collagen II showed that the expression levels of P16 and cleaved caspase-3 in the IDD and IDD+shRNA-NC groups increased, and that of collagen II decreased significantly, indicating that NP cell senescence and apoptosis increased significantly during IDD, and ECM catabolism increased. These degenerative changes were significantly reduced in the IDD+shRNA-BRD4 group (Figures 7(d)–7(h)). In summary, BRD4 inhibition reduced the senescence and apoptosis of NP cells and increased ECM anabolism, thereby improving IDD *in vivo*.

## 3. Discussion

Severe IDD in middle-aged and older individuals can lead to a decline in the quality of life and chronic disability [5, 9, 12]. However, the mechanisms of IDD development are unclear. Therefore, it is important to clarify the occurrence and development of IDD and identify novel therapeutic targets. Senescence and apoptosis are the main causes of IDD [10, 27, 28]. Replicative senescence is a phenomenon in which telomeres are shortened due to continuous division of cells, which induces senescence [29, 30]. Prolonged replicative senescence of IVD cells may be linked to IDD. However, this process may be accelerated by, for instance, inflammation, oxidative stress, mitochondrial dysfunction, and mechanical load) [3, 13]. As IDD progresses, the levels of proinflammatory factors—including IL-1*α*, IL-1*β*, IL-6, IL-17, and TNF-*α*—increase significantly in degenerative IVD. These elicit a local autoimmune inflammatory response, promoting IVD cell senescence, apoptosis, and ECM catabolism, resulting in dysfunction and structural changes of the IVD [3, 31–33]. Therefore, antisenescence and antiapoptosis therapy of IVD cells may delay IDD.

BRD4 is a member of the bromodomain and extraterminal protein family and regulates apoptosis, senescence, inflammation, transcription and the cell cycle [34–38]. Zhou et al. [17] reported that BRD4 inhibition blocks glial activation by inhibiting inflammation and apoptosis, ameliorating ischemic brain damage. Wang et al. [63] reported that BRD4 downregulation may regulates MAPK and NF-*κ*B signaling and activate autophagy to suppress MMP-13 expression in diabetic IDD. BRD4 inhibition alleviates ECM degradation by suppressing NLRP3 inflammasome activity through nuclear factor-kappa B signaling pathway in NP cells [64]. However, it is remains unclear whether BRD4 is involved in the development of IDD, and a role for BRD4 in the regulation of NP cells senescence and apoptosis during IDD has not been reported. We evaluated the BRD4 level of NP tissues from patients with IDD; the severity of IVD degeneration was positively correlated with the BRD4 level. BRD4 expression was highest in patients with grade V IDD, indicating that an increased BRD4 level aggravates IDD. Also, BRD4 expression was increased in senescent human NP cells. The P53, P21 and P16 levels and SA-*β*-gal activity were assayed to assess the level of senescence. Inhibition of BRD4 reduced the levels of these markers in NP cells undergoing replicative senescence.

The level of IL-1*β* is increased in degenerative IVD, accelerates the senescence of neighbouring cells, increases inflammation, and triggers IVD cell apoptosis [32]. IL-1*β* also forms a positive feedback loop to stimulate the release of other inflammatory mediators and the synthesis of MMPs, enhancing local catabolism in IVD [3]. *In vitro*, IL-6, IL-8, and IL-17 levels increased significantly after stimulating human NP and AF cells with IL-1*β*. Therefore, IL-1*β* initiates the inflammatory cascade by promoting the release of IL-6, IL-8 and IL-17 [39, 40]. In this study, we used IL-1*β* to induce NP cells and so simulate the pathophysiology of IDD. IL-1*β* increases the level of senescence and apoptosis of NP cells. As expected, inhibition of BRD4 significantly reduced the levels of senescence- and apoptosis-related proteins.

Autophagy refers to the self-digestion of cells using lysosomes to degrade their own damaged, denatured or senescent macromolecular substances and organelles under the influence of external environmental factors [41, 42]. Autophagy is a self-protection mechanism of eukaryotic cells and is important for cell survival and death [43]. Many age-related degenerative diseases show enhanced autophagy, such as osteoarthritis, cardiovascular diseases, neurodegenerative diseases and diabetes [44–46]. Autophagy is closely related to aging and apoptosis [41, 47]. Ma et al. [48] showed that autophagy controls bone marrow-derived mesenchymal stem cell senescence during bone aging. Chen et al. [13] used 3-MA to inhibit autophagy and eliminated the antiapoptotic and senescence effects of SIRT6 on NP cells. Kornicka et al. [49] reported that 5-azacytydine and resveratrol via modulation of autophagy reverse senescence and apoptosis of adipose stem cells. BRD4 reduces apoptosis induced by oxidative stress after spinal cord injury by regulating autophagy [50]. Mu et al. [20] showed that BRD4 inhibits autophagy mediated by PINK1/Parkin to prevent high-fat diet-induced diabetic cardiomyopathy. Therefore, we hypothesised that BRD4 exerts a powerful antiapoptotic and antisenescence effect by activating autophagy. We use the expression levels of LC3, Beclin1, P62 and ATG5 as typical markers of autophagy. TEM showed that BRD4 inhibition activates autophagy in NP cells. The classical autophagy inhibitor 3-MA reversed the antisenescence and antiapoptotic effects of BRD4. Therefore, BRD4 inhibition slows the progression of IDD by activating autophagy. The physiological function of IVD depends on the molecular composition of NP ECM, principally collagen II and aggrecan [51]. During the progression of IDD, the gradual loss of collagen II and aggrecan due to NP ECM metabolic disorder leads to a decrease in IVD height, loss of the boundary between the AF and NP, and a decline in the ability to withstand mechanical loads [51]. MMPs and ADAMTSs are the main catabolic enzymes in IVD. MMP3 and ADAMTS4 are the main ECM degradative enzymes in NP cells, and their expression is increased in denatured IVD [52, 53]. The inflammatory factor IL-1*β* is an important catabolism inducer, which is significantly upregulated in IDD and promotes its progression by inhibiting the expression of genes related to ECM synthesis and increasing that of genes encoding ECM-degrading enzymes ^3^. We found that inhibition of BRD4 in NP cells significantly reduced the IL-1*β*-induced increase in MMP3 and ADAMTS4 levels and reversed the IL-1*β*-induced decrease in the expression of collagen II and aggrecan. These protective effects of BRD4 were reversed by 3-MA, indicating that the protective effect of BRD4 is mediated by autophagy.

Autophagy is mediated by a series of complex signaling networks, most of which enter the AMPK/mTOR/ULK1 signaling pathway [54]. AMPK is a key energy sensor that can directly phosphorylate ULK1 to initiate autophagy; by contrast, mTOR is an evolutionarily highly conserved serine/threonine protein kinase, which triggers autophagy by inhibiting the negative regulator ULK1. AMPK activation negatively regulates mTOR and thereby increases autophagy activity [55]. Autophagy is triggered by the phosphorylation of ULK1 (Atg1), which coordinates the interactions of other key proteins in the autophagy cascade [56]. Melatonin activates autophagy via the AMPK/mTOR/ULK1 signaling pathway to reduce vascular calcification [54]. Curcumin induces autophagy and enhances autophagy flux in an AMPK/mTOR/ULK1-dependent manner, thereby attenuating mitochondrial dysfunction induced by oxidative stress in NP cells [56]. Interestingly, our results showed that the AMPK/mTOR/ULK1 signaling pathway was activated by inhibition of BRD4. To determine whether autophagy activation depends on AMPK, we pretreated NP cells with CC (AMPK inhibitor). CC reversed not only the increase of p-AMPK and p-ULK1 mediated by BRD4 inhibition but also the decrease of p-mTOR. Also, CC significantly reduced autophagy activation of NP cells after BRD4 inhibition. These findings indicate that the AMPK/mTOR/ULK1 pathway is involved in BRD4-mediated autophagy activation.

Although our study proved the prodenaturation effect of BRD4 in NP cells, our study still has some limitations. First, in addition to NP, the progression of IDD is also related to AF or CEP degeneration. Therefore, in future research, other types of cells involved in the IDD process should be further studied. Secondly, we only silenced BRD4 in NP cells by siRNA to study its effect on NP cell degeneration. Extracellular BRD4 and unknown binding receptors may also be involved in IDD. Therefore, this is also one of the directions of future research. Third, this study confirmed that BRD4 affects the apoptosis and senescence of NP cells through AMPK/mTOR/ULK1 signaling pathways, but it is not clear whether other signaling pathways are involved in the IDD process. Therefore, it is necessary to conduct in-depth research on the role and mechanism of BRD4 in the IDD process in future research. In addition, although the rat model used in our study can simulate the pathological development of IDD to a certain extent, the IVD of rodents is different from mammals in terms of function and biomechanical properties, and monkeys and pigs need to be involved in future research. Or the IDD model of goat for further study.

**Figure 8:**
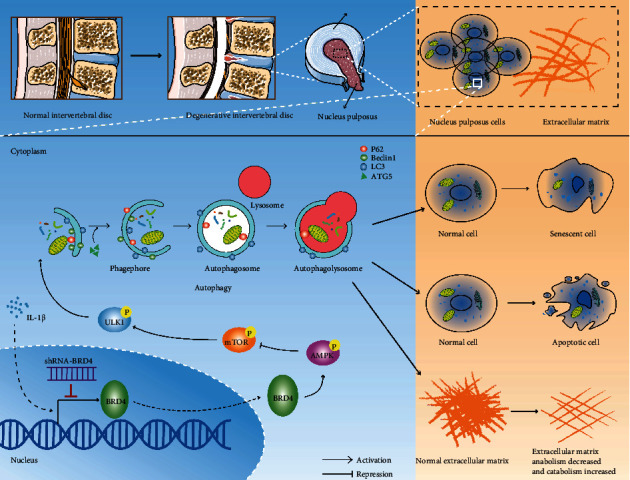
Inhibition of BRD4 induces AMPK/mTOR/ULK1 signaling to modulate autophagy-associated senescence and apoptosis of NP cells.

In conclusion, BRD4 increased with the severity of IDD in human IVD NP tissues. *In vitro*, BRD4 inhibition inhibited the senescence and apoptosis of NP cells by regulating the AMPK/mTOR/ULK1 pathway to activate autophagy, thereby delaying IDD (Figure 8). The rat annulus-needle puncture model confirmed the protective effect of BRD4 inhibition on IDD *in vivo*. Our findings indicate that BRD4 has therapeutic potential for IDD.

## 4. Materials and Methods

### 4.1. Collection of Human NP Tissue

All procedures performed in research involving human participants complied with the ethical standards of the institution and/or the National Research Council and complied with the 1964 Declaration of Helsinki and its subsequent amendments or similar ethical standards. All experimental protocols were approved by the Ethics Committee of the Lanzhou University Second Hospital, and written informed consent was obtained from each patient. Normal NP tissue (grade I) was collected from patients who underwent idiopathic scoliosis surgery and lumbar spondylolysis (*n* = 15; 9 males; 6 females; age 12–20 years, mean 15.2 years), and degenerated NP tissues from patients undergoing discectomy and spinal fusion surgery (*n* = 24; 17 males; 7 females; age 21–68 years, mean 42.7 years) (grade II–V). The patients did not have a history of tumour or of tuberculosis or other infectious diseases. The degree of degradation of human NP tissue samples was classified using the Pfirrmann grade ^60, 61^. NP tissue was removed during lumbar NP surgery and transported to the laboratory.

### 4.2. Human NP Cell Culture and Transfection

Normal NP tissue specimens were rinsed thoroughly in PBS to remove visible blood cells and mixed fibrous annulus tissue, cut into 1 mm^2^ pieces, and digested with 0.25 mg/mL collagenase II (Proteintech, China) for 8 h and centrifuged at 800 rpm for 5 min to collect the precipitate. Dulbecco's modified Eagle's medium comprised Nutrient Mixture F-12 (DMEM/F-12) (Gibco, Thermo Fisher Scientific) with 10% foetal bovine serum (FBS; Gibco, Thermo Fisher Scientific) and 1% antibiotics (100 U/mL penicillin and 100 U/mL streptomycin) (Gibco) at 37°C in a 5% CO_2_ incubator. The medium was refreshed every 2–3 days. Cell growth was visualised under a light microscope. When the cells reached 80–90% confluence, they were digested with 0.25% trypsin-ethylenediaminetetraacetic acid (Gibco, Thermo Fisher Scientific), and passaged at a 1 : 2 ratio. Passage 2 cells were used in subsequent experiments. In addition, BRD4 shRNA or scrambled shRNA were purchased from Genechem (Shanghai, China). Lentiviruses were transfected at a multiplicity of infection of 100 in serum-free DMEM. After 24 h, the supernatant was replaced with complete medium (DMEM containing 10% FBS). Transfection efficacies were detected by western blotting and RT-PCR, and the cells were further cultured for 3 days, and passaged for subsequent experiments.

### 4.3. Haematoxylin-Eosin Staining

When the cells covered about 80% of the coverslip, the slide was washed with PBS. The cells were fixed with 4% paraformaldehyde for 20 min, washed three times with PBS, nuclei were stained with haematoxylin for 8 min, and the cells were washed with tap water. Next, the cells were differentiated with 1% acidic alcohol solution for 30 s and rinsed with tap water. The cells were foamed in saturated lithium carbonate solution for 30 s and rinsed with tap water. Cells were counterstained in eosin solution for 1 min, rinsed with tap water and digitally scanned using an EVOS microscope (EVOS-FL Cell Imaging System, Thermo Fisher Scientific).

### 4.4. Toluidine Blue Staining

When the cells covered about 80% of the coverslip, we performed toluidine blue staining. The coverslip was washed three times with PBS and cells were fixed with 95% alcohol for 15 s and washed three times with PBS. Next, toluidine blue solution was added for 5 min, and an equal volume of distilled water was added over the next 15 min. The cells were rinsed with tap water, dried, glycerin-sealed sheet, and digitally scanned using an EVOS microscope (EVOS-FL Cell Imaging System, Thermo Fisher Scientific).

### 4.5. Safranin-O Staining

When the cells covered about 80% of the coverslip, we performed Safranin-O staining. The coverslip was washed with PBS, cells were stained with haematoxylin for 10 min, and washed three times with PBS. Next, cells were stained with 2% Fast Green for 3 min, decolourised with 1% HCl-alcohol for 5 min, stained with 0.1% Safranin-O for 5–10 min and washed twice with PBS, and digitally scanned using an EVOS microscope (EVOS-FL Cell Imaging System, Thermo Fisher Scientific).

### 4.6. Senescence-Associated *β*-Galactosidase Staining

According to the instructions of the manufacturer, the Senescence-associated *β*-Galactosidase (SA-*β*-gal) Staining Kit (Beyotime, Shanghai, China) was used to assay senescence. NP cells were fixed at room temperature for 15 min and washed twice with PBS. Next, we added 1 mL of *β*-galactosidase staining solution (10 *μ*L of staining solution A, 10 *μ*L of staining solution B, 930 *μ*L of staining solution C, and 50 *μ*L of X-Gal solution) to the wells and transferred the plates to an incubator (no CO_2_) at 37°C at least overnight. Under an inverted light microscope (EVOS-FL Cell Imaging System, Thermo Fisher Scientific), aged NP cells with high SA-*β*-gal activity were blue.

### 4.7. Immunofluorescence Staining

A total of 1 × 10^6^ cells were seeded on coverslips in a six-well plate. Cells were fixed with 4% neutral paraformaldehyde for 20 min, washed three times in PBS, and permeabilised in 0.5% Triton X-100 for 20 min. The cells were washed three times with PBS and blocked (10% goat serum) for 1 h. Next, the cells were incubated with the primary antibody overnight at 4°C followed by a fluorescein isothiocyanate- or cyanine 3-conjugated secondary antibody at 37°C for 1 h. The cells were rinsed three times in PBS for 5 min each, 4′,6-diamidino-2-phenylindole (DAPI, Invitrogen) was applied to counterstain nuclei, and the cells were incubated for 10 min with a chromogen. Finally, the cells were photographed under a fluorescence microscope (Olympus Inc., Tokyo, Japan).

### 4.8. Hoechst 33258 Staining

NP cells were stained with Hoechst 33258 staining solution (Solaribo, Beijing, China), and enumerated in a blinded manner as described previously [59]. NP cells on coverslips were washed with PBS and fixed with 4% paraformaldehyde for 20 min. After washing three times with PBS, cells were stained with Hoechst 33258 for 15 min in the dark. After washing the cells with PBS, a fluorescence microscope (Olympus Inc., Tokyo, Japan) was used to detect nuclear morphological changes indicative of apoptosis. During apoptosis, the NP cell nucleus is dense and shows dense staining, or is dense and densely stained in fragments, and the staining is bright. We counted the number of apoptotic cells and the total number of cells and calculated the apoptosis rate as

(1) $$\begin{array}{c} \text{Apoptosis rate }\left(\%\right)=\left(\frac{N_{\text{apoptotic cells}}}{N_{\text{total cells}}}\;\right)\times 100. \end{array}$$

### 4.9. Terminal Deoxynucleotidyl Transferase dUTP Nick-End Labelling Staining

The level of DNA damage was assessed by terminal deoxynucleotidyl transferase (TdT) dUTP nick-end labelling (TUNEL) staining. In brief, a total of 1 × 10^6^ cells were seeded on a coverslip in a six-well plate. Cells were fixed with 4% neutral paraformaldehyde for 20 min, incubated in 3% H_2_O_2_ and 0.1% Triton X-100 for 10 min, and washed with PBS three times. Finally, according to the manufacturer's instructions, cells were stained using the In Situ Cell Death Detection Kit (Roche, Basel, Switzerland) for 60 min at 37°C and nuclei were stained with DAPI at 37°C. Images were captured under a fluorescence microscope (Olympus Inc., Tokyo, Japan). TUNEL-positive cells were counted by two independent and blinded observers, and the apoptosis rate was calculated as

(2) $$\begin{array}{c} \text{Apoptosis rate }\left(\%\right)=\left(\frac{N_{\text{TUNEL}‐\text{positive cells}}}{N_{\text{total cells}}}\right)\times 100. \end{array}$$

### 4.10. RNA Extraction and Real-Time PCR

Total RNA was obtained from cultured NP tissue samples or NP cells with TRIzol Reagent (Qiagen; Valencia, CA). According to the manufacturer's instructions, using a cDNA reverse transcription kit (TaKaRa; Dalian, China) we synthesised complementary DNA (cDNA) from 1 *μ*g of total RNA. The mRNA level was determined by reverse transcription quantitative polymerase chain reaction (RT-PCR) according to standard procedures using the Light Cycler 96 System (Roche; Mannheim, Germany). mRNA levels were quantified with TB green (TB Green Supermix; TaKaRa) and normalised to that of GAPDH. Thermocycling conditions were as follows: 1 cycle at 95°C for 30 sec, 40 cycles at 95°C for 5 sec, 40 cycles at 60°C for 30 sec, 1 cycle at 95°C for 5 sec, 1 cycle at 60°C for 1 min, and then 1 cycle at 50°C for 30 sec. The primers for RT-PCR were as follows: *Homo* BRD4—forward 5′-ACCTCCAACCCTAACAAGCC-3′ and reverse 5′-TTTCCATAGTGTCTTGAGCACC-3′, and *Homo* GAPDH—forward 5′-TCAAGAAGGTGGTGAAGCAGG-3′ and reverse 5′-TCAAAGGTGGAGGAGTGGGT-3′. GAPDH was used for normalisation, and the experiments were performed in triplicate. Data were analysed by the 2^−ΔΔCt^ method.

### 4.11. Western Blotting

NP tissues and cells were washed three times with cold PBS and lysed in radioimmunoprecipitation assay buffer (Beyotime, Shanghai, China). Tissue and cell lysates were centrifuged at 4°C and 12,000 rpm for 20 min, and the supernatant was collected. The Bradford method was used to assay protein concentrations [60]. An equal amount of protein was separated by 10% sodium dodecyl sulphate-polyacrylamide gel electrophoresis and transferred to a polyvinylidene fluoride (PVDF) membrane (Millipore; Billerica, MA). At room temperature, the membrane was blocked in 5% skimmed milk powder for 2 h and incubated overnight at 4°C with BRD4 (1 : 100) (Santa Cruz, Dallas, TX), P16 (1 : 1000), P21 (1 : 1000), P53 (1 : 1000), p-P53 (1 : 1000) (Cell Signaling Technology, Danvers, MA), LC3 (1 : 1000), Beclin1 (1 : 1000), P62 (1 : 1000) (Proteintech, Wuhan, China), Bcl-2 (1 : 1000), Bax (1 : 1000), cleaved caspase-3 (1 : 1000), mTOR (1 : 1000), p-mTOR (1 : 1000), AMPK (1 : 1000), p-AMPK (1 : 1000), ULK1 (1 : 1000), p-ULK1 (1 : 1000), collagen II (1 : 1000), MMP3 (1 : 1000), aggrecan (1 : 1000), and ADAMTS4 (1 : 1000) (Affinity Biosciences, Zhenjiang, China) primary antibodies. On day 2, the PVDF membranes were washed three times in Tris-buffered saline containing 0.05% Tween-20 (TBST). The membranes were next incubated with the corresponding secondary antibodies at room temperature (22–28°C) for 2 h. The *β*-actin expression level was used as the internal control. Protein bands were detected using an enhanced chemiluminescence kit and ImageJ (ver. 1.52q, https://www.imagej.en.softonic.com/) software was used to process band intensities. The experiments were performed as three independent replicates.

### 4.12. Transmission Electron Microscopy

Human NP cells were fixed in 2.5% (*w*/*v*) glutaraldehyde overnight and postfixed in 2% (*w*/*v*) osmium tetroxide for 1 h, followed by staining with 2% (*w*/*v*) uranyl acetate for 1 h. After a series of acetone dehydration treatments, samples were embedded in Araldite, cut into semi-thin sections, stained with toluidine blue, and examined under a Hitachi transmission electron microscope (Hitachi, Tokyo, Japan).

### 4.13. Animal Experiments

Adult male Sprague–Dawley rats (200–220 g; Animal Center of Lanzhou University, Lanzhou, China) were reared under a 12 h light/dark cycle at a standard temperature and were provided food and water *ad libitum*. The rat annulus-needle puncture IDD model was generated as described previously [13]. The rats were randomly divided into the sham, IDD, IDD+shRNA-NC, and IDD+shRNA-BRD4 groups. Rats were sedated with isoflurane and anesthetized with pentobarbital (40 mg/kg via intraperitoneal injection). Under sterile conditions, a small incision was made in the sagittal skin to expose the Co7/8 intervertebral disc, and a syringe needle (21 G) was inserted into the NP parallel to the CEP (from the dorsal to the ventral side). To ensure consistent needle penetration, needle length was determined according to the AF and NP dimensions (~5 mm measured in preliminary experiments). The needles were rotated 360° and retained in the IVD for 1 min. To eliminate the influence of injection volume, microliter syringes (10 *μ*L, Gaoge, Shanghai, China) were used to inject the lentivirus-normal control (shRNA-NC) or lentivirus-BRD4-RNAi (shRNA-BRD4) into the central space of NP tissue. The rat was returned to the cage to continue feeding. The operators were blinded to the experimental group assignments.

### 4.14. Magnetic Resonance Imaging

The T2-weighted (T2W) MRI sequence is a simple and reliable method for monitoring water proton dipolar interaction in the ECM of NP tissue [61]. Magnetic resonance imaging (MRI) was performed 8 weeks after surgery using a 3.0 T clinical magnet (Siemens, Erlangen, Germany) to evaluate the signal and structural changes in sagittal T2-weighted images. T2W sections were obtained using the following settings: a fast-spin echo sequence with a time-to-repetition (TR) of 5400 ms and a time-to-echo (TE) of 920 ms; 320 (h) 9256 (v) matrix; a field-of-view of 260°; and four excitations. The section thickness was 2 mm with a gap of 0 mm. The Pfirrmann grading of disc degeneration is as follows [57, 58]: Grade I, homogeneous IVD with bright high-intensity white signal and normal disc height; grade II, uneven IVD, with or without horizontal bands, and high-intensity white signal; grade III, uneven IVD of slightly reduced height, with medium grey signal intensity; grade IV, uneven IVD of moderately reduced height, with grey or black signal strength; grade V, uneven IVD with high black signal strength and a collapsed IVD space. Finally, the rats were euthanised and the IVDs were collected for histological and immunohistochemical analyses.

### 4.15. Histological Examination

At week 8 after the operation, the rats were euthanised and the caudal IVD tissue (Co7/8) was separated, fixed with pentobarbital, decalcified with 10% ethylenediaminetetraacetic acid, and embedded in paraffin for serial sectioning. The sections were stained with haematoxylin and eosin (HE), and the histological grades of the specimens were determined to quantify damage based on a previously described method [62]. For immunohistochemical analysis, the tissue sections were deparaffinised in xylene and rehydrated in an ethanol concentration gradient. After antigen retrieval and blocking solution treatment, the slides were incubated with primary antibodies (BRD4, P16, cleaved caspase-3, and collagen II) at 4°C overnight. Next, the sections were incubated with the secondary antibody (Biological Technology; Wuhan, China) for 30 min. Finally, after washing twice with PBS, 3′,3-diaminobenzidine tetrahydrochloride (DAB) solution was added for 5 min, and the section was counterstained with haematoxylin and visualised by optical microscopy.

### 4.16. Data Analysis

All statistical analyses were performed with the GraphPad Prism software (ver. 8.0). The Shapiro-Wilk test was used to inspect the normality and homogeneity of variance of all the data. For experimental protocols with only two groups, an unpaired Student's *t*-test was performed with normally distributed data and Mann–Whitney nonparametric test with other data. For experiments with more than two groups, statistical significance was determined using one-way ANOVA for continuous variables. Pearson correlation coefficient test was performed to evaluate the relationship between BRD4 expression and Pfirrmann grade. Data are presented as mean ± standard of multiple (3–4 times) repeated experiments with 3–4 samples per experiment. *P* < 0.05 was considered indicative of significance.

## Data Availability

The original contributions presented in the study are included in the article. Further inquiries can be directed to the corresponding author.

## Supplementary Materials

Supplementary Materials Supplementary Figure 1: identification of human primary NP cells in culture. (A–C) Morphology of P0, P1, and P2 NP cells; mostly long fusiform, polygonal, irregular, or star-shaped; magnification: 200x. (D) HE staining—human NP cells were long fusiform, polygonal, or other irregular shapes; magnification, 200x. (E) Safranin-O staining—nuclei of human NP cells stained dark red, and the patina was lightly stained; magnification, 200x. (F) Toluidine blue staining—human NP cells stained blue; magnification, 400x. (G–I) Blue fluorescence, DAPI (nuclei); green fluorescence collagen II and aggrecan; red fluorescence, Sox9; magnification, 400x.

## References

[1] Yang S., Zhang F., Ma J., Ding W. (2020). Intervertebral disc ageing and degeneration: the antiapoptotic effect of oestrogen. *Ageing Research Reviews*.

[2] Liu Y., Qu Y., Liu L. (2019). PPAR-*γ* agonist pioglitazone protects against IL-17 induced intervertebral disc inflammation and degeneration *via* suppression of NF-*κ*B signaling pathway. *International Immunopharmacology*.

[3] Zhang G. Z., Deng Y. J., Xie Q. Q. (2020). Sirtuins and intervertebral disc degeneration: roles in inflammation, oxidative stress, and mitochondrial function. *Clinica Chimica Acta*.

[4] Ohnishi T., Sudo H., Tsujimoto T., Iwasaki N. (2018). Age-related spontaneous lumbar intervertebral disc degeneration in a mouse model. *Journal of Orthopaedic Research*.

[5] Yu L., Hao Y., Xu C., Zhu G., Cai Y. (2019). LINC00969 promotes the degeneration of intervertebral disk by sponging miR-335-3p and regulating NLRP3 inflammasome activation. *IUBMB Life*.

[6] Feng Y., Egan B., Wang J. (2016). Genetic factors in intervertebral disc degeneration. *Genes & Diseases*.

[7] Henry N., Clouet J., Le Bideau J., Le Visage C., Guicheux J. (2018). Innovative strategies for intervertebral disc regenerative medicine: from cell therapies to multiscale delivery systems. *Biotechnology Advances*.

[8] Xi Y., Ma J., Chen Y. (2020). PTEN promotes intervertebral disc degeneration by regulating nucleus pulposus cell behaviors. *Cell Biology International*.

[9] Penolazzi L., Lambertini E., Bergamin L. S. (2018). MicroRNA-221 silencing attenuates the degenerated phenotype of intervertebral disc cells. *Aging*.

[10] Chen D., Xia D., Pan Z. (2016). Metformin protects against apoptosis and senescence in nucleus pulposus cells and ameliorates disc degeneration *in vivo*. *Cell Death & Disease*.

[11] Ding L., Zeng Q., Wu J. (2017). Caveolin-1 regulates oxidative stress-induced senescence in nucleus pulposus cells primarily via the p53/p21 signaling pathway in vitro. *Molecular Medicine Reports*.

[12] Song J., Wang H. L., Song K. H. (2018). CircularRNA 104670 plays a critical role in intervertebral disc degeneration by functioning as a ceRNA. *Experimental & Molecular Medicine*.

[13] Chen J., Xie J. J., Jin M. Y. (2018). Sirt6 overexpression suppresses senescence and apoptosis of nucleus pulposus cells by inducing autophagy in a model of intervertebral disc degeneration. *Cell Death & Disease*.

[14] Scrivo A., Bourdenx M., Pampliega O., Cuervo A. M. (2018). Selective autophagy as a potential therapeutic target for neurodegenerative disorders. *Lancet Neurology*.

[15] Dikic I., Elazar Z. (2018). Mechanism and medical implications of mammalian autophagy. *Nature Reviews. Molecular Cell Biology*.

[16] Luo R., Liao Z., Song Y. (2019). Berberine ameliorates oxidative stress-induced apoptosis by modulating ER stress and autophagy in human nucleus pulposus cells. *Life Sciences*.

[17] Zhou Y., Gu Y., Liu J. (2019). BRD4 suppression alleviates cerebral ischemia-induced brain injury by blocking glial activation via the inhibition of inflammatory response and pyroptosis. *Biochemical and Biophysical Research Communications*.

[18] Lin S., Du L. (2020). The therapeutic potential of BRD4 in cardiovascular disease. *Hypertension Research*.

[19] Sanders Y. Y., Lyv X., Zhou Q. J. (2020). Brd4-p300 inhibition downregulates Nox4 and accelerates lung fibrosis resolution in aged mice. JCI Insight 5. *JCI Insight*.

[20] Mu J., Zhang D., Tian Y., Xie Z., Zou M. H. (2020). BRD4 inhibition by JQ1 prevents high-fat diet-induced diabetic cardiomyopathy by activating PINK1/Parkin-mediated mitophagy in vivo. *Journal of Molecular and Cellular Cardiology*.

[21] Jing X., Shao S., Zhang Y. (2020). BRD4 inhibition suppresses PD-L1 expression in triple-negative breast cancer. *Experimental Cell Research*.

[22] Jiang Y., Zhu L., Zhang T. (2017). BRD4 has dual effects on the HMGB1 and NF-*κ*B signalling pathways and is a potential therapeutic target for osteoarthritis. *Biochimica et Biophysica Acta - Molecular Basis of Disease*.

[23] An Q. D., Li Y. Y., Zhang H. X. (2018). Inhibition of bromodomain-containing protein 4 ameliorates oxidative stress-mediated apoptosis and cartilage matrix degeneration through activation of NF-E2-related factor 2-heme oxygenase-1 signaling in rat chondrocytes. *Journal of Cellular Biochemistry*.

[24] He J., Zhang A., Song Z. (2019). The resistant effect of SIRT1 in oxidative stress-induced senescence of rat nucleus pulposus cell is regulated by Akt-FoxO1 pathway. *Bioscience Reports*.

[25] Rodrigues-Pinto R., Richardson S. M., Hoyland J. A. (2014). An understanding of intervertebral disc development, maturation and cell phenotype provides clues to direct cell-based tissue regeneration therapies for disc degeneration. *European Spine Journal*.

[26] Zhang Y., Yang X., Ge X., Zhang F. (2019). Puerarin attenuates neurological deficits via Bcl-2/Bax/cleaved caspase-3 and Sirt3/SOD2 apoptotic pathways in subarachnoid hemorrhage mice. *Biomedicine & Pharmacotherapy*.

[27] Zhang Z., Lin J., Nisar M. (2019). The Sirt1/P53 axis in diabetic intervertebral disc degeneration pathogenesis and therapeutics. *Oxidative Medicine and Cellular Longevity*.

[28] Cherif H., Bisson D. G., Mannarino M., Rabau O., Ouellet J. A., Haglund L. (2020). Senotherapeutic drugs for human intervertebral disc degeneration and low back pain. *eLife*.

[29] Sanokawa-Akakura R., Akakura S., Ostrakhovitch E. A., Tabibzadeh S. (2019). Replicative senescence is distinguishable from DNA damage-induced senescence by increased methylation of promoter of rDNA and reduced expression of rRNA. *Mechanisms of Ageing and Development*.

[30] Hernandez-Segura A., Nehme J., Demaria M. (2018). Hallmarks of cellular senescence. *Trends in Cell Biology*.

[31] Wang K., Chen T., Ying X. (2019). Ligustilide alleviated IL-1*β* induced apoptosis and extracellular matrix degradation of nucleus pulposus cells and attenuates intervertebral disc degeneration *in vivo*. *International Immunopharmacology*.

[32] Wang Y., Che M., Xin J., Zheng Z., Li J., Zhang S. (2020). The role of IL-1*β* and TNF-*α* in intervertebral disc degeneration. *Biomedicine & Pharmacotherapy*.

[33] Ruiz-Fernández C., Francisco V., Pino J. (2019). Molecular relationships among obesity, inflammation and intervertebral disc degeneration: are adipokines the common link?. *International Journal of Molecular Sciences*.

[34] Wang H., Fu H., Zhu R. (2020). BRD4 contributes to LPS-induced macrophage senescence and promotes progression of atherosclerosis-associated lipid uptake. *Aging (Albany NY)*.

[35] Zuo H., Wang S., Feng J., Liu X. (2019). BRD4 contributes to high-glucose-induced podocyte injury by modulating Keap1/Nrf2/ARE signaling. *Biochimie*.

[36] Dey A., Yang W., Gegonne A. (2019). BRD4 directs hematopoietic stem cell development and modulates macrophage inflammatory responses. *The EMBO Journal*.

[37] Zhu W., Wu R. D., Lv Y. G., Liu Y. M., Huang H., Xu J. Q. (2020). BRD4 blockage alleviates pathological cardiac hypertrophy through the suppression of fibrosis and inflammation via reducing ROS generation. *Biomedicine & Pharmacotherapy*.

[38] Wen X., Klionsky D. J. (2017). BRD4 is a newly characterized transcriptional regulator that represses autophagy and lysosomal function. *Autophagy*.

[39] Akyol S., Eraslan B. S., Etyemez H., Tanriverdi T., Hanci M. (2010). Catabolic cytokine expressions in patients with degenerative disc disease. *Turkish Neurosurgery*.

[40] Yang W., Yu X. H., Wang C. (2015). Interleukin-1*β* in intervertebral disk degeneration. *Clinica Chimica Acta*.

[41] Kang C., Elledge S. J. (2016). How autophagy both activates and inhibits cellular senescence. *Autophagy*.

[42] Shirakabe A., Ikeda Y., Sciarretta S., Zablocki D. K., Sadoshima J. (2016). Aging and autophagy in the heart. *Circulation Research*.

[43] Saha S., Panigrahi D. P., Patil S., Bhutia S. K. (2018). Autophagy in health and disease: a comprehensive review. *Biomedicine & Pharmacotherapy*.

[44] Lian W. S., Ko J. Y., Wu R. W. (2018). MicroRNA-128a represses chondrocyte autophagy and exacerbates knee osteoarthritis by disrupting Atg12. *Cell Death & Disease*.

[45] Ren J., Zhang Y. (2018). Targeting autophagy in aging and aging-related cardiovascular diseases. *Trends in Pharmacological Sciences*.

[46] Chu C. T. (2019). Mechanisms of selective autophagy and mitophagy: implications for neurodegenerative diseases. *Neurobiology of Disease*.

[47] Zhan S., Wang K., Xiang Q. (2020). lncRNA HOTAIR upregulates autophagy to promote apoptosis and senescence of nucleus pulposus cells. *Journal of Cellular Physiology*.

[48] Ma Y., Qi M., An Y. (2018). Autophagy controls mesenchymal stem cell properties and senescence during bone aging. *Aging Cell*.

[49] Kornicka K., Szłapka-Kosarzewska J., Śmieszek A., Marycz K. (2019). 5-Azacytydine and resveratrol reverse senescence and ageing of adipose stem cells via modulation of mitochondrial dynamics and autophagy. *Journal of Cellular and Molecular Medicine*.

[50] Li Y., Xiang J., Zhang J., Lin J., Wu Y., Wang X. (2020). Inhibition of Brd4 by JQ1 promotes functional recovery from spinal cord injury by activating autophagy. *Frontiers in Cellular Neuroscience*.

[51] Long J., Wang X., Du X. (2019). JAG2/Notch2 inhibits intervertebral disc degeneration by modulating cell proliferation, apoptosis, and extracellular matrix. *Arthritis Research & Therapy*.

[52] Liu S., Yang S. D., Huo X. W., Yang D. L., Ma L., Ding W. Y. (2018). 17*β*-Estradiol inhibits intervertebral disc degeneration by down-regulating MMP-3 and MMP-13 and up-regulating type II collagen in a rat model. *Artificial Cells, Nanomedicine, and Biotechnology*.

[53] Russo F., Ambrosio L., Ngo K. (2019). The role of type I diabetes in intervertebral disc degeneration. *Spine (Phila Pa 1976)*.

[54] Chen W. R., Yang J. Q., Liu F., Shen X. Q., Zhou Y. J. (2020). Melatonin attenuates vascular calcification by activating autophagy via an AMPK/mTOR/ULK1 signaling pathway. *Experimental Cell Research*.

[55] Sun L., Zhao M., Liu A. (2018). Shear stress induces phenotypic modulation of vascular smooth muscle cells via AMPK/mTOR/ULK1-mediated autophagy. *Cellular and Molecular Neurobiology*.

[56] Kang L., Xiang Q., Zhan S. (2019). Restoration of autophagic flux rescues oxidative damage and mitochondrial dysfunction to protect against intervertebral disc degeneration. *Oxidative Medicine and Cellular Longevity*.

[57] Pfirrmann C. W., Metzdorf A., Zanetti M., Hodler J., Boos N. (2001). Magnetic resonance classification of lumbar intervertebral disc degeneration. *Spine (Phila Pa 1976)*.

[58] Urrutia J., Besa P., Campos M. (2016). The Pfirrmann classification of lumbar intervertebral disc degeneration: an independent inter- and intra-observer agreement assessment. *European Spine Journal*.

[59] Wang S., Zhang H., Geng B. (2018). 2-Arachidonyl glycerol modulates astrocytic glutamine synthetase via p38 and ERK1/2 pathways. *Journal of Neuroinflammation*.

[60] Bradford M. M. (1976). A rapid and sensitive method for the quantitation of microgram quantities of protein utilizing the principle of protein-dye binding. *Analytical Biochemistry*.

[61] Jin L. Y., Lv Z. D., Wang K. (2018). Estradiol alleviates intervertebral disc degeneration through modulating the antioxidant enzymes and inhibiting autophagy in the model of menopause rats. *Oxidative Medicine and Cellular Longevity*.

[62] Han B., Zhu K., Li F. C. (2008). A simple disc degeneration model induced by percutaneous needle puncture in the rat tail. *Spine*.

[63] Wang J., Hu J., Chen X. (2019). BRD4 inhibition regulates MAPK, NF-*κ*B signals, and autophagy to suppress MMP-13 expression in diabetic intervertebral disc degeneration. *The FASEB Journal*.

[64] Hong J., Li S., Markova D. Z. (2020). Bromodomain-containing protein 4 inhibition alleviates matrix degradation by enhancing autophagy and suppressing NLRP3 inflammasome activity in NP cells. *Journal of Cellular Physiology*.

