# Supplementary figures and images for: BRD4 Inhibition Suppresses Senescence and Apoptosis of Nucleus Pulposus Cells by Inducing Autophagy during Intervertebral Disc Degeneration: An In Vitro and In Vivo Study
Source: Oxid Med Cell Longev. 2022 Mar 11;2022:9181412. doi: 10.1155/2022/9181412 (PMC8933081; doi:10.1155/2022/9181412)

Supplementary Fig. 1.


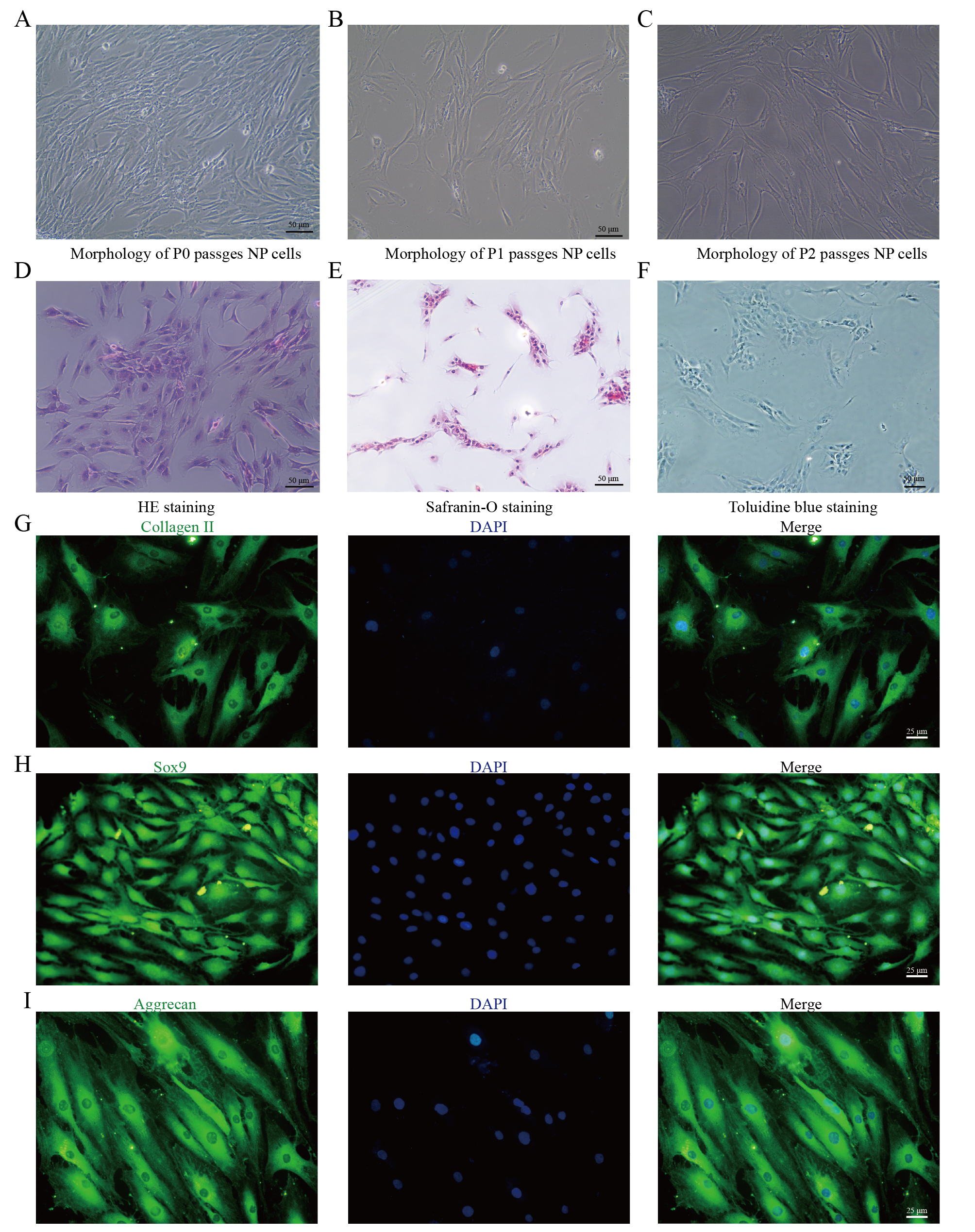

Supplement: Supplementary Materials — Supplementary Figure 1: identification of human primary NP cells in culture. (A–C) Morphology of P0, P1, and P2 NP cells; mostly long fusiform, polygonal, irregular, or star-shaped; magnification: 200x. (D) HE staining—human NP cells were long fusiform, polygonal, or other irregular shapes; magnification, 200x. (E) Safranin-O staining—nuclei of human NP cells stained dark red, and the patina was lightly stained; magnification, 200x. (F) Toluidine blue staining—human NP cells stained blue; magnification, 400x. (G–I) Blue fluorescence, DAPI (nuclei); green fluorescence collagen II and aggrecan; red fluorescence, Sox9; magnification, 400x. [file 9181412.f1.docx]
